# Ureteral stenosis due to extraskeletal chondroma: An exceptionally rare case report

**DOI:** 10.1016/j.eucr.2025.103209

**Published:** 2025-09-15

**Authors:** Toshimitsu Tohya, Hiroshi Ishizaki, Yoshiki Futakuchi, Hideyuki Fukui, Kazutaka Ozono, Munekage Yamaguchi

**Affiliations:** a Department of Obstetrics and Gynecology, Kumamoto General Hospital, Japan; b Department of Urology, Kumamoto General Hospital, Japan; c Department of Surgical Pathology, Kumamoto General Hospital, Japan; d Department of Obstetrics and Gynecology, Faculty of Life Sciences, Kumamoto University, Japan

## Abstract

We report an exceptionally rare case of ureteral stenosis caused by an extraskeletal chondroma. A woman in her 60s underwent laparoscopic hysterectomy for presumed uterine fibroid torsion, but later developed severe right flank pain. Imaging revealed hydronephrosis and ureteral stricture suspicious for tumor. Ureteroscopy with biopsy confirmed chondroma, representing only the second reported case worldwide after 1973. The patient has been managed with periodic ureteral stent exchanges, leading to symptom relief and improved hydronephrosis. This case highlights the extreme rarity of ureteral chondroma, the diagnostic challenge of differentiating it from malignancy, and the importance of histopathological confirmation.

## 1 Introduction

Chondroma is typically a benign tumor arising in bone tissue, composed of chondrocytes, and most commonly develops in the epiphyseal regions of the long bones of the extremities. Occurrence in the trunk is rare, with reported sites including the dura mater, pharynx, oral cavity, and skin.^1^ Chondroma arising in the urinary tract is extremely uncommon; among reported cases, almost all involve the bladder, with eight cases described to date, all in women.^2^ Here, we report a case of extraskeletal chondroma compressing and narrowing the right ureter. To our knowledge, only one similar case has been documented in the literature, published in 1973.^3^

## 2 Case presentation

**Fig. 1:**
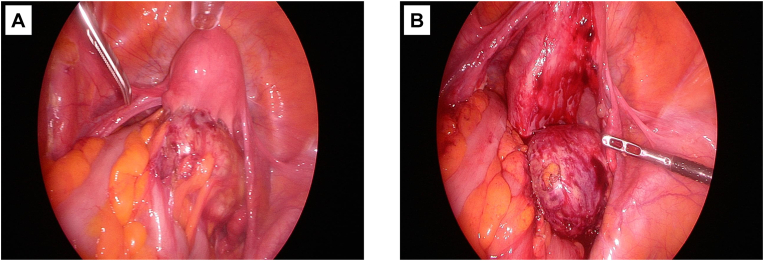
**Macroscopic intraoperative findings of the uterus** (A) A pedunculated subserosal fibroid was identified on the posterior uterine wall, showing dense adhesions to the sigmoid colon. (B) Adhesiolysis was carefully performed, and inflammatory changes of the surrounding peritoneum were suspected.

The patient was a woman in her 60s, gravida 0 para 0, who presented with lower abdominal pain and abdominal distension. Upper and lower gastrointestinal endoscopy at a local hospital revealed no abnormalities; however, abdominal CT identified a 7-cm subserosal uterine fibroid, and she was referred to our department. Transvaginal ultrasonography and pelvic MRI confirmed a 7-cm subserosal fibroid along with several intramural fibroids measuring 2–3 cm in diameter. As torsion of the subserosal fibroid could not be excluded, total laparoscopic hysterectomy with bilateral salpingo-oophorectomy (TLH + BSO) was performed. Intraoperatively, a pedunculated subserosal fibroid was found on the posterior uterine wall, with adhesions to the surrounding peritoneum and sigmoid colon (Fig. 1). Adhesiolysis was performed, the bilateral posterior leaves of the broad ligament were dissected, and the course of the ureters was identified to complete the surgery. Within the range observable during laparoscopy, no gross abnormalities were detected in the ureters, and the kidneys were not inspected intraoperatively.

**Fig. 2:**
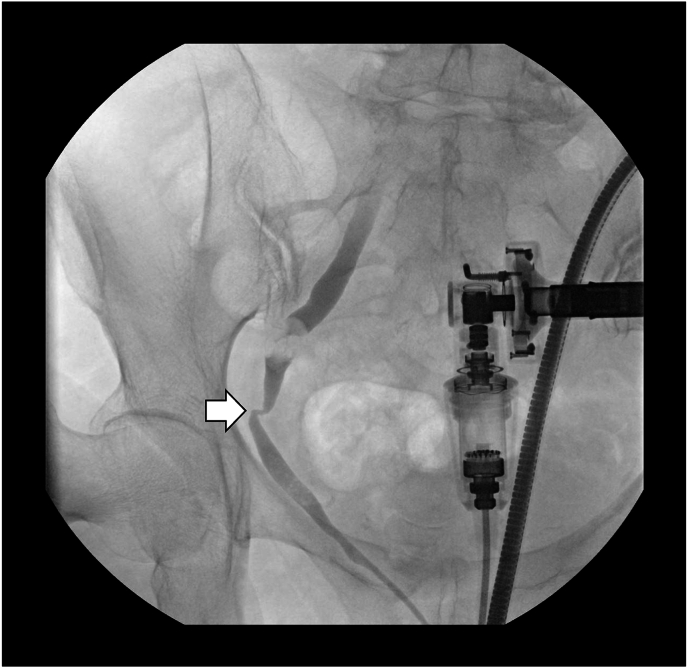
**Retrograde pyelography following laparoscopic hysterectomy** Contrast study revealed compression of the right ureter with associated obstruction (arrow), performed after the patient presented with right flank pain.

**Fig. 3:**
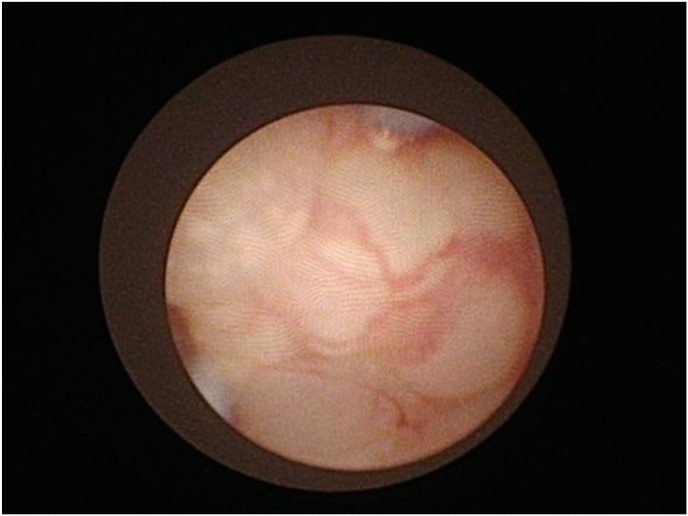
**Ureteroscopic findings of the right urete**r At the stenotic site, the ureteral mucosa appeared edematous. A biopsy was obtained from the same location.

**Fig. 4:**
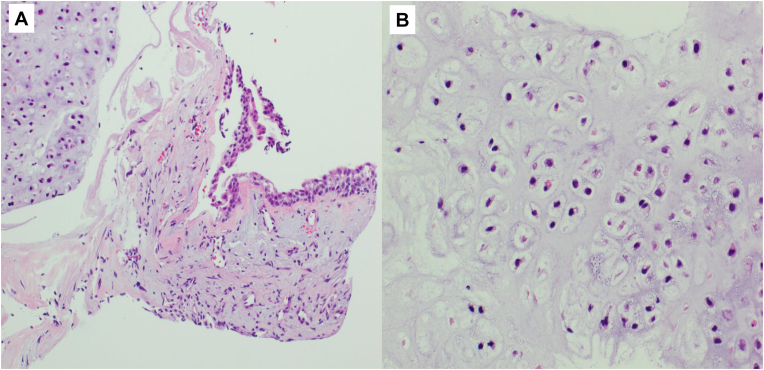
**Histopathological features of the right ureteral biopsy** (A) Well-circumscribed lesion composed of mature hyaline cartilage located in the submucosa of the right ureter (haematoxylin and eosin, × 100). (B) Higher magnification showing mature hyaline cartilage without cytological atypia, mitotic activity, or necrosis (haematoxylin and eosin, × 200).

Postoperatively, the patient's pain subsided and she was discharged. However, approximately one week after discharge, she developed severe right flank pain. Contrast-enhanced CT revealed right hydronephrosis, raising suspicion of ureteral injury or ligation. Retrograde pyeloureterography performed by the urology team demonstrated compressive ureteral stenosis, with ureteral tumor as a possible diagnosis (Fig. 2). Subsequent ureteroscopic examination (Fig. 3) and biopsy of the stenotic site were carried out, and histopathology confirmed a chondroma (Fig. 4). The patient is currently managed with a right ureteral stent, which is exchanged periodically, resulting in improvement of the hydronephrosis. The patient was offered further definitive surgery consisting of partial resection of the right ureteral tumor site with end-to-end anastomosis; however, possibly because the pain had subsided, the patient is hesitant to undergo the procedure.

## 3 Discussion

In this report, we describe a case of chondroma compressing and narrowing the right ureter. To our knowledge, only one similar case has been reported in the literature, published in 1973.^3^ Chondroma is typically a benign tumor arising from bone tissue, most commonly located in the epiphyseal regions of the long bones of the extremities. Although rare, they can also develop in the trunk. Within the urinary tract, approximately eight cases of bladder chondroma have been reported, all in female patients.^2^

The etiology of chondroma remains unclear, but genetic factors have been implicated. Studies on hereditary multiple exostoses have demonstrated associations with specific gene mutations, such as exostosin glycosyltransferase 1 (EXT1) and exostosin glycosyltransferase 2 (EXT2), and a positive family history may increase risk.^4^ Trauma and chronic inflammation have also been proposed as potential contributors.^5^ In addition, adipose tissue harbors multipotent progenitor cells capable of differentiating into osteoblasts and chondrocytes, suggesting that chondromas might arise from such pluripotent cells.^6^ In this case, there was no family history of chondroma, and imaging studies revealed no findings suggestive of multiple chondromas. Although the tumor might have arisen as a result of mechanical manipulation during ureteral dissection at surgery, the interval between surgical handling of the ureter and the onset of chondroma was too short, making a traumatic etiology unlikely. Given the presence of severe pelvic adhesions, the most plausible explanation is that chronic inflammation served as the underlying cause.

There are no established diagnostic criteria or guidelines for chondroma, and diagnosis must be made on a case-by-case basis. Clinically, differentiation between chondroma and chondrosarcoma is crucial. Imaging with CT or MRI is essential; MRI findings can be helpful in distinguishing benign from malignant lesions. However, imaging alone cannot reliably differentiate chondroma from chondrosarcoma, and histopathological examination remains indispensable for definitive diagnosis.^7^ While only one case of ureteral chondroma has been reported,^3^ several cases of ureteral chondrosarcoma have been described.^7^ Most extraskeletal chondrosarcomas occur in the extremities, with only 10–13 % arising in the retroperitoneum, abdomen, or pelvis.^8^ Primary renal or ureteral chondrosarcoma is exceedingly rare and considered an exceptional entity.^9^ Because accurate diagnosis has direct implications for treatment and prognosis, precise histological evaluation supported by appropriate immunohistochemistry is essential for identifying mesenchymal chondrosarcoma.^7^

As chondroma is a benign tumor, observation is generally appropriate in asymptomatic cases.^10^ When symptoms are present, surgical excision of the tumor may be required. This patient has been advised to undergo partial resection of the right ureter at the tumor site with end-to-end anastomosis, and surgery may be performed if the patient provides consent.

## CRediT authorship contribution statement

**Toshimitsu Tohya:** Writing – original draft, Visualization, Project administration, Methodology, Investigation, Funding acquisition, Formal analysis, Data curation, Conceptualization. **Hiroshi Ishizaki:** Resources. **Yoshiki Futakuchi:** Resources. **Hideyuki Fukui:** Resources. **Kazutaka Ozono:** Resources. **Munekage Yamaguchi:** Writing – review & editing, Visualization, Validation, Supervision, Data curation.

## Informed consent

Informed consent was obtained from the patient for publication of this case report.

## Conflicts of interest

The authors declare no conflicts of interest.
